# Lobbying, fast and slow: principles of influence beyond reason

**DOI:** 10.1057/s41309-025-00255-9

**Published:** 2025-11-21

**Authors:** Iskander De Bruycker

**Affiliations:** Maastricht University, Maastricht, Netherlands

**Keywords:** Lobbying, Influence, Interest groups, Persuasion, Cognitive bias

## Abstract

This research agenda article presents a novel perspective on lobbying influence by distinguishing between two modes of lobbying: fast and slow. Fast lobbying targets intuitive, heuristic-based decision-making, while slow lobbying engages deliberate, effortful cognitive processes among policymakers. The prevailing interest group literature has largely focused on the latter, conceptualizing lobbying as a rational, evidence-driven exchange. However, this article argues that lobbying influence extends beyond conscious deliberation to include subconscious persuasion. It reinterprets psychological insights—particularly Robert Cialdini’s seven principles of influence—as tools for understanding how intuitive persuasion shapes elite decision-making. While these principles are widely recognized in behavioral economics and marketing, their implications for political influence remain underexplored. The article examines the extent to which such mechanisms have been integrated into interest group research and highlights areas that remain neglected. It bridges cognitive and social psychology with interest group research to conceptualize fast lobbying as a distinct and underexamined mode of influence. The article concludes by outlining a research agenda and raising ethical questions about the role of intuitive persuasion in democratic policymaking.

## Introduction

Mark McGann served as Uber’s top lobbyist between 2014 and 2016. In July 2022 he stood at the center of the so called “Uber files.” These leaked documents expose how McGann secretly met with European top politicians, such as Emmanuel Macron, Boris Johnson and Neelie Kroes. McGann appears to have been a good friend of the then-Minister of Mobility for Brussels, Pascal Smet. MacGann was criticized as having exploited that friendship when Uber wanted to roll out taxi services in Brussels.^1^ This emblematic example captures the stereotypical view of lobbying as driven by informal ties.

Academic literature typically contrasts with such stereotypes, framing lobbying as a rational, evidence-based exchange of resources between interest organizations and policymakers (Aizenberg and Müller 2021; Bunea and Baumgartner 2014; Dür & Mateo, 2012; Klüver 2013; Rasmussen and Gross 2015; Stevens and Willems 2024). This perspective largely overlooks informal, relational, or intuitive sources of influence. Yet policymakers, like all humans, are boundedly rational and often rely on cognitive shortcuts and personal relationships that shape their decisions beyond pure logic (Jones 1999). Some studies note how long-term interactions and insider status shape perceptions of interest group influence (Albareda et al. 2023; Braun 2013). This article similarly challenges the assumption that lobbying outcomes result solely from calculated reasoning.

The article proposes a new conceptual framework to better understand how lobbying works through both rational and intuitive routes. Specifically, it introduces the distinction between “fast” and “slow” lobbying—modeled on dual-process theories of decision-making—to capture how policymakers respond not only to information and evidence, but also to emotional, relational, and heuristic cues. As a research agenda article, its aim is to theorize these modes of influence and inspire future studies to explore the intuitive, affective dynamics of lobbying more systematically. At the same time, it invites reflection on the ethical and normative dimensions of lobbying. While lobbying is often seen as a legitimate contribution to policymaking through the provision of expertise, recognizing its more intuitive, relational side challenges this technocratic view and raises ethical questions about how influence is exercised—and justified—in democratic settings.

The terminology “fast” and “slow” lobbying draws inspiration from psychologist and Nobel laureate Daniel Kahneman, who uses “thinking fast and slow” to refer to System 1 and System 2 decision making processes. System 1 involves intuitive, effortless, and emotional decision-making, typically associated with the limbic system of the brain. In contrast, System 2 refers to a mode of decision-making that is conscious, effortful, calculated, and deliberative, primarily linked to the prefrontal cortex (Kahneman 2011; Nazir and Liljenstrom 2015). Most academic discussions on lobbying influence assume that policymakers engage in System 2 reasoning, where lobbying efforts focus on providing information, expertise, and strategic support to inform rational deliberations. I term this approach lobbying “slow.”

However, Kahneman notes that much of human decision-making is driven by System 1, where intuition and emotions guide choices, often with System 2 later rationalizing these decisions. As Kahneman (2011) observes, System 2 “often endorses or rationalizes ideas and feelings that were generated by System 1.” This perspective aligns with findings in political psychology and governance (e.g., Jones 2001; Versluis et al. 2025; Vis 2019), which show that policymakers, like other individuals, are heavily influenced by heuristics and intuitive decision-making. By focusing solely on deliberate, instrumental exchanges of information, existing studies may overlook an important dimension of lobbying: its ability to engage with policymakers’ intuitive, automatic decision-making processes. To fully understand lobbying influence, we must account for the dynamics of “fast” lobbying—those that tap into the subconscious, intuitive, and emotional aspects of elite decision-making. This research agenda article seeks to unpack these “fast” lobbying dynamics, exploring how they differ from traditional “slow” lobbying approaches and examining their potential to shape policymaker behavior. Its aim is not to make an empirical contribution, but to inspire future studies on interest group lobbying to also tap into the affective, relational, and intuitive dimensions of policymaking that have so far remained underexplored. Specifically, it asks: *What constitutes “fast” and “slow” lobbying*,* and how do these modes influence the behavior of policymakers?*

“Fast” lobbying refers to efforts aimed at influencing heuristic and intuitive decision-making among policymakers. It seeks to benefit from psychological and emotional processes, such as familiarity, relationships, and comfort. Fast lobbying is not necessarily quicker than slow lobbying but aims to influence quick, instinctive, and automatic decision-making. By contrast, “slow” lobbying focuses on influencing complex, deliberate reasoning, planning, and rational evaluations by policymakers and their teams. In short, fast lobbying targets System 1 reasoning among policymakers—their intuitive, automatic, and emotionally driven responses—while slow lobbying targets System 2 reasoning among policymakers, characterized by conscious, deliberate, and analytical processing.

Rational reasoning and intuitive responses are interconnected and often overlap, so lobbying strategies targeting one often engage the other. Fast and slow lobbying usually operate simultaneously rather than sequentially, with their relative importance depending on the policymaker and context. For example, long-standing relationships—often built through slow lobbying—can foster trust that makes fast, intuitive influence more effective in high-pressure situations. This interplay, and the blurred boundaries between lobbying types, parallels Kahneman’s idea of fast and slow thinking as “fictitious characters”—conceptual tools rather than strict categories. I adopt these concepts to understand lobbying influence, recognizing that fast and slow approaches often coexist and jointly shape policymaker behavior. This dynamic interplay will be a recurring theme throughout the article.

To explore the mechanisms through which fast lobbying operates, this article draws on psychological research on persuasion—particularly the work of Cialdini (2021), whose seven principles of influence have become foundational in understanding how intuitive, heuristic-based decisions are shaped. Cialdini’s framework offers a holistic and systematic overview of persuasion techniques, drawn from decades of empirical research and real-world strategies used by compliance professionals such as salespeople, negotiators, and marketers. Although widely recognized in domains like marketing and behavioral science, these techniques have not yet been systematically applied to lobbying. For this reason, the article adopts Cialdini’s principles as a practical and theoretically grounded lens to conceptualize fast lobbying and to inspire further empirical research in this underexplored area.

## Principle 1: reciprocity

One of the most fundamental principles illustrating the interplay of fast and slow lobbying is *reciprocity*. This principle shows how lobbying efforts engage both rational, calculated exchanges and intuitive, emotional responses. At its core, reciprocity involves offering benefits with the expectation of receiving comparable returns (Molm 2010). In essence, it means repaying what one has received with comparable goods or actions (Cialdini 2021). Reciprocity is a pervasive norm in human interaction and serves as a fundamental rule of conduct (Melamed et al. 2020), contributing not only to utilitarian exchanges but also to the development of trust and solidarity (Molm et al. 2007).

Thus, reciprocity is not solely driven by rational calculation; it also encompasses emotional and symbolic dimensions. Psychological research highlights both its instrumental value—enhancing predictability of returns—and its symbolic value, which fosters trust and emotional bonds (Molm et al. 2007). This distinction mirrors the difference between slow and fast lobbying. Slow lobbying emphasizes the instrumental value of reciprocity, aiming for rational, mutually beneficial exchanges. Fast lobbying, by contrast, taps into the symbolic value, appealing to ingrained social norms and emotional instincts to foster goodwill and trust.

Instrumental reciprocity is central to lobbying scholarship, particularly within resource exchange theory, a dominant framework in interest group studies (Bouwen 2004; Chalmers 2013; Colli and Reykers 2023; Eising 2007; Fraussen et al. 2015; Tallberg et al. 2018). This theory posits that lobbying influence is shaped by the exchange of resources—primarily expertise and political support—between interest groups and policymakers (Binderkrantz et al. 2014; Klüver 2013). Policymakers reciprocate by granting access and influence. These exchanges align with slow lobbying, as they presume goal-oriented, rational reasoning.

However, reciprocity also has a symbolic dimension, leading to integrative outcomes such as trust, emotional bonds, and solidarity (Molm 2010). Research shows that actors in reciprocal exchanges express stronger emotional regard and commitment than those in purely negotiated transactions (Molm et al. 2000). These relationships are perceived as more unified and grounded in mutual respect. This symbolic dimension aligns with fast lobbying, which seeks to cultivate emotional and psychological ties rather than transactional exchanges. Fast lobbying leverages subconscious responses—such as familiarity, obligation, and personal connection—that create fertile ground for informal influence.

The symbolic nature of reciprocity is particularly evident in lobbying practices involving gifts, favors, and sponsorships. For example, Roberta Metsola, President of the European Parliament, attended a lavish gastronomic event hosted by the Confrérie des Chevaliers du Tastevin, where she was honored as a “dame.”^2^ She later declared 142 gifts, ranging from champagne and chocolates to gold coins.^3^ While many of these gifts do not directly relate to policy, they cater to personal needs and thus exemplify fast lobbying. Such gestures can help lobbyists attract attention and generate goodwill, potentially opening the door to more substantive, evidence-based exchanges typical of slow lobbying.

Viewing reciprocity through the lens of fast lobbying reveals a significant research gap. First, interest group research should acknowledge the non-transactional outcomes of resource exchange, such as trust, goodwill, and loyalty. Second, more attention is needed on exchange goods that lack clear policy value but serve personal needs—particularly gifts and favors. Even small gifts can trigger feelings of loyalty that may influence decision-making. Third, reciprocity should be examined as a long-term relationship-building tool, not just a mechanism for influencing single policy issues. Ongoing exchanges can cultivate friendships and trust-based alliances that shape policymaker behavior over time.

## Principle 2: authority

which suggests that people comply more readily with perceived experts or authoritative figures. In this article, authority is not understood in the classical political science sense of having formal, institutional power to make binding decisions (e.g. see Zürn et al. 2012). Rather, it refers to *perceived authority*: the symbolic, epistemic, or professional legitimacy that prompts heuristic acceptance of influence (Bushman 1984). This conception of authority focuses on how people respond to signals of competence and credibility. Cialdini cites the Milgram (1963) experiment—where participants obeyed harmful orders from an authority figure—to shows how perceived authority can override judgment and elicit intuitive compliance.

While lobbyists lack formal decision-making power, they can strategically project perceived authority. A common strategy is to present themselves as experts. Research shows that messages from perceived authorities are more persuasive, as people tend to trust sources they view as knowledgeable (Stehr and Grundmann 2011). Lobbyists typically assert authority in three domains: by emphasizing specialized expertise (technical, legal, scientific, or policy-related), aligning with broad constituencies, and highlighting economic relevance (Flöthe 2020; Halpin and Fraussen 2017; Hanegraaff and De Bruycker 2020). These strategies appeal to both slow, deliberative reasoning and fast, intuitive processing.

At the EU level, interest groups clearly use this strategy in their online self-presentations. For example, Transport & Environment emphasizes its epistemic authority by stating on their website: “*We strongly believe in the power of science and evidence in policymaking. Our team is made up of experts in the field, and every piece of evidence we put out is thoroughly reviewed to ensure accuracy*.”^4^ In public affairs, a lobby group’s credibility and reputation are essential for influence (McGrath 2006). Here, credibility refers to the perceived reliability and expertise of a lobbyist at a given point in time, while reputation reflects a more durable, accumulated perception of credibility across repeated interactions. Both feed into the perception of authority that policymakers may respond to intuitively. Building a reputation requires consistent delivery of reliable input and long-term relationship-building. Scandals in EU lobbying involving Volkswagen, Uber, Amazon, or Huawei show how quickly reputations can be damaged.

Studies suggest policymakers prefer trusted partners with proven expertise (Bernhagen 2013; Hanegraaff and De Bruycker 2020). Factors such as staff size, organizational age, and membership base affect a group’s perceived authority (Flöthe 2020; McKay 2012; Stevens and De Bruycker 2020). Lobbyists boost authority by forming coalitions or citing respected sources. For instance, after Mario Draghi published a high-profile report on EU competitiveness in 2024, several lobby groups aligned their messaging with his findings to boost credibility.^5^

As Cialdini notes, titles can also subconsciously shape how people assess credibility. In lobbying, the term “lobbyist” is often avoided due to negative connotations. Instead, more appealing titles like “public affairs expert” or “government relations officer” are used to signal professionalism and expertise (Eiselt et al. 2025). The involvement of CEOs in lobbying can further enhance authority. For example, Sundar Pichai, CEO of Google, publicly advocated for responsible AI regulation in the Financial Times, lending weight to broader discussions around the EU’s AI Act.^6^

Leveraging authority is a hallmark of fast lobbying, as it increases message impact by invoking heuristics rather than reasoned judgment. Research shows that when expert advice is provided, brain regions responsible for decision-making become less active, suggesting that individuals “offload” judgment to the expert (Meshi et al. 2012). Similarly, policymakers may accept lobbyists’ arguments not for their content, but because of the perceived authority of the source. This reflects how lobbyists often function as informal service bureaus for policymakers and their staff, with their input sometimes being directly incorporated—almost verbatim—into legislative texts (Hall and Deardorff 2006).^7^

That said, a policymaker’s openness to authoritative input is not purely intuitive. Authority is cultivated over time through consistent delivery of quality work, integrity, and relationship-building. It is not merely the result of superficial cues like titles or self-presentation. A lobbyist’s credibility often stems from slow lobbying efforts—providing expert information and political support over time.

Ultimately, the principle of authority spans both fast and slow lobbying. There is a dynamic interplay between the two: the reputational capital built through slow lobbying can later amplify the impact of fast, heuristic-based influence. Organizations like Transport & Environment may build authority through a track record of reliable input. Once established, such authority enables policymakers to quickly recognize and trust the messenger, even in high-pressure or low-information situations. However, authority is fragile. Missteps can quickly erode credibility, forcing lobbyists to rebuild trust. More attention should be paid to how lobbyists mobilize authority cues—whether by positioning themselves as experts or aligning with respected figures—and how these cues shape policymaker behavior.

## Principle 3: scarcity

Cialdini principle discussed here is scarcity, which holds that people are more likely to desire or protect something when they perceive it as scarce, threatened, or at risk of being lost. Scarcity enhances perceived value by signaling limited availability. A classic example cited by Cialdini (2021) is the cookie experiment by Freedman and Fraser (1966), where participants rated cookies from a smaller batch as more desirable than identical ones from a larger batch—demonstrating how scarcity inflates subjective value. While cookies may be trivial in lobbying, the scarcity principle plays a significant role in public affairs. Lobbyists often frame issues as perceived threats to policy opportunities, economic advantages, or social goods—portraying them as rare, fleeting, or in danger of vanishing. Urgency, in this context, serves as a rhetorical device that activates the underlying scarcity mechanism. By conveying that a window of opportunity is closing—or that inaction may lead to irreversible losses—lobbyists can evoke fast, emotionally charged reactions.

A recent BusinessEurope press release illustrates this strategy: *“There is a real danger that businesses*,* and in particular energy-intensive industries*,* relocate outside of Europe permanently… To prevent further production losses*,* the EU state aid framework must be further adjusted…”*^8^

Here, urgency is the framing (“real danger”, “relocate outside of Europe”), while scarcity is the underlying perceived loss (jobs, production capacity). To clarify, scarcity and urgency are not conceptually equivalent but as in the example are often used in tandem. Scarcity refers to the underlying perception of limitedness or potential loss, while urgency is one way of activating this perception. Lobbyists use urgency as a narrative tool to stimulate intuitive responses associated with scarcity.

Cialdini (2021) notes that the persuasive power of scarcity draws on two well-established psychological theories associated with fast thinking: prospect theory and psychological reactance theory. Prospect theory (Kahneman and Tversky 2013) suggests that individuals are more motivated to avoid losses than to pursue equivalent gains. This “loss aversion” means that lobbying messages framed around potential setbacks (e.g., job losses or missed investments) are likely to provoke stronger emotional responses than gain-framed equivalents. Despite its clear relevance, the role of loss aversion in lobbying remains underexplored.

Psychological reactance theory holds that people become motivated to act when they perceive their freedom of choice as threatened. Lobbying appeals that emphasize “last chances” or “closing windows” exploit this mechanism. For instance, a 2024 BusinessEurope statement framed policy timing as crucial: *““A new*,* in-depth study published today by BusinessEurope*,* developed with economic consultancy Compass Lexecon*,* shows that a more competitive energy and climate transition is still possible but only if swift action is taken by EU legislators during the next EU cycle.”*^9^ Such language implicitly suggests that future options may disappear, evoking fear of lost agency or missed opportunity. However, Cialdini (2021) warns that reactance can backfire if individuals perceive manipulation. This resistance can be mitigated through techniques promoting transparency and choice. Presenting balanced information, including potential drawbacks, fosters trust and transparency (see Eisend 2006). As noted by Cialdini (2021) and demonstrated empirically by Guéguen et al. (2013), simply stating “You are free to decide” increases compliance. Lobbyists may enhance credibility by acknowledging trade-offs and reinforcing policymakers’ autonomy—for example, admitting short-term costs while emphasizing long-term policy benefits.

Scarcity-driven lobbying aligns with fast lobbying because it relies on heuristic responses: fear of missing out, protection of valued assets, and avoidance of regret. However, when backed by credible evidence—such as economic forecasts or impact studies—scarcity appeals can also serve slow lobbying by enabling more reasoned judgment. For example, the BusinessEurope example above cites “an in-depth study,” blending urgency with empirical substantiation. In sum, scarcity is a powerful persuasive tool in lobbying. Future research could examine how urgency is strategically constructed to activate scarcity perceptions, how loss aversion frames affect policymaker behavior, and how resistance to pressure can be mitigated by techniques that emphasize autonomy, credibility, and transparency.

## Principle 4: liking

In the context of lobbying, likability may similarly enhance influence. One EU official informally noted that lobbyists from a particular large technology firm were perceived as especially pleasant and nice to work with.^10^. This illustrates how personal charm and social ease can contribute to lobbying effectiveness, even if the mechanisms behind likability are complex and context-dependent. Cialdini (2021) identifies several techniques through which likability can be enhanced, many of which resonate with lobbying practices.

One key factor is *relational closeness*. Friendship is a powerful route to influence: people are more receptive to requests from those they consider friends. Cialdini famously describes how Tupperware parties exploit this dynamic. In lobbying, close personal ties can similarly reduce resistance. For example, Uber lobbyist Mark McGann’s friendly relationship with Brussels Minister of Mobility Pascal Smet likely facilitated smoother interactions. Even indirect connections—such as being referred by a mutual acquaintance—can generate trust and goodwill. Repeated positive contact reinforces this effect: familiarity tends to increase liking. While public affairs professionals often change positions, long-term engagements between the same lobbyists and policymakers can allow trust to accumulate over time.

Likability also grows through perceived *similarity and social mirroring*. People are drawn to those who share their background, values, or experiences. Lobbyists may emphasize shared education, ideology, or even hobbies to build rapport. It is common practice to pair lobbyists with policymakers of the same nationality or linguistic group. Research shows that lobbying organizations are more likely to engage legislators when their representatives share the same gender or national background (Bunea et al. 2025; De Bruycker 2024; Hollman and Murdoch 2018). Compliments, too, can play a subtle role. Even when perceived as insincere, praise can increase likability and reinforce desired behavior (Grant et al. 2010). For instance, hospitality-sector groups publicly praised government COVID-19 relaxation measures, not out of gratitude alone, but to help consolidate and extend those policies (De Bruycker 2025). Physical attractiveness, though not yet studied in public affairs, influences perceptions through the halo effect—where attractive individuals are seen as more competent, intelligent, and trustworthy (Jackson et al. 1995). Such appearance-based biases may subtly affect which lobbyists are heard or given attention.

Another pathway to likability is *positive association and context*. Cialdini’s “luncheon technique” illustrates how people become more receptive when messages are delivered over lunch or diner. Kahneman (2011, p. 43) similarly notes that judges were more likely to grant parole after meal breaks, as Danziger et al. (2011) found favorable rulings dropped sharply within a session and returned to about 65% following a break. (Danziger et al. 2011). Lobbyists often capitalize on this by hosting informal meetings over food or drinks, as Jack Abramoff famously did in his Washington restaurants.^11^ Despite how common this practice is, empirical research on its effectiveness remains limited. Relatedly, likability can be transferred via association with respected figures. Greenpeace has used celebrities like Jane Fonda and Jason Momoa to promote its goals^12^, while business lobbyists often rely on former politicians—such as Nick Clegg for Meta or José Manuel Barroso for Goldman Sachs—whose prior reputations may enhance credibility and access. Though research on “revolving door” lobbying has focused on informational advantages (e.g., Egerod et al. 2024; LaPira and Thomas 2014), interpersonal likability may also contribute to revolvers’ effectiveness.

These techniques to improve liking clearly do not aim to stimulate analytical reasoning but rather to shape intuitive, subconscious approval. As such, the principle of liking aligns primarily with fast lobbying, engaging System 1 thinking. Emotional connections—through friendship, similarity, or positive associations—can influence perceptions and judgments without requiring cognitive effort. However, it is important to recognize that many elements of liking, particularly friendship and familiarity, often develop through slow lobbying efforts involving sustained and repeated interactions over time. These slow lobbying activities cultivate trust and rapport, which then enable fast, intuitive acceptance during lobbying encounters. While liking may not directly contribute to slow lobbying in the moment of influence, slow lobbying frequently lays the relational groundwork that makes liking-based fast lobbying effective. In this way, liking acts as both an entry point for influence, initiated through intuition, and a potential outcome of long-term relationship-building that supports deeper engagement. In sum, liking plays a significant yet underappreciated role in lobbying. Future research should explore how liking and friendship shape access and influence, and assess the effectiveness of tactics aimed at enhancing likability.

## Principle 5: social proof

Cialdini’s principle of social proof holds that individuals are strongly influenced by the “wisdom of the crowd,” meaning the behavior and opinions of others, often aligning with what is perceived as popular or socially desirable. In lobbying research, social proof is often conceptualized as a form of currency in transactional exchanges, where public support and political information act as social cues offered in return for access or influence (Berkhout 2013; Bevan and Rasmussen 2020; Halpin and Fraussen 2017; Rasmussen et al. 2018). Empirical studies show that lobbying organizations with broad public backing are more likely to achieve their policy goals. Policymakers interpret public support as a valid cue, making them more receptive to lobbyists whose positions align with majority sentiment (Rasmussen et al. 2018; Willems and Beyers 2023). Lobbyists can amplify this effect by referencing public support or using publicly spirited strategies (Hanegraaff et al. 2016; Junk 2016). However, the influence of public support is context-dependent. Cialdini (2021) identifies three conditions that enhance the power of social proof: uncertainty, the many, and similarity.

*Uncertainty* arises when decision-makers lack expertise or clarity, making them more reliant on social cues. This is especially relevant for policymakers unfamiliar with a policy domain or working on complex policy issues. Research shows that undecided policymakers are more open to persuasion (Hojnacki and Kimball 1998). While committee members may be well-informed, other parliamentarians voting in plenary may rely on cues from party colleagues or ideologically aligned lobbyists. In such cases, aligning with the socially endorsed position may feel like the safest or most legitimate option.

The second condition, *the many*, suggests that the more people who support a position, the more valid and acceptable it appears. Lobbyists are more likely to succeed when their positions are backed by strong public opinion or are highly visible in media debates (Rasmussen et al. 2018). This also explains bandwagoning in lobbying: when lobbyists see others mobilizing around an issue, they may join in to avoid missing out on influencing a high-profile debate (Baumgartner and Leech 2001; Halpin 2011). This behavior reinforces momentum and amplifies the perceived legitimacy of certain policy issues.

The third condition, *similarity*, increases the persuasive power of social proof by enhancing the perceived relevance of others’ behavior. Policymakers are more influenced by cues from individuals or groups they perceive as similar—ideologically, demographically, or professionally. For instance, right-wing legislators are more likely to support business groups when backed by right-wing public opinion, while left-wing legislators respond more to civil society groups endorsed by left-leaning publics (De Bruycker and Rasmussen 2021). Social proof is thus more persuasive when it comes from perceived in-group sources.

Importantly, social proof can be manufactured. As the Cambridge Analytica scandal revealed, Facebook data were exploited to micro-target voters with emotionally charged messages, fragmenting audiences and distorting perceptions of collective opinion. Through such techniques, campaigns can prompt social validation and amplify the appearance of popular momentum.^13^ Similarly, lobbyists can construct social proof by creating the appearance of broad societal backing, even when actual support is limited. While some tactics may be benign, others raise concerns about democratic legitimacy.

One strategy that overlaps with the authority principle is forming broad, heterogeneous coalitions. By aligning with diverse organizations, lobbyists signal cross-sectoral support, enhancing credibility and suggesting consensus (Beyers and De Bruycker 2018; Junk 2019). Another tactic is the use of public interest frames or representative claims, invoking broad constituencies like “European citizens” or “taxpayers” to imply broad support (Flöthe 2020; Raknes and Ihlen 2020). Empirical research shows that such framing increases lobbying success (De Bruycker 2019). A more controversial tactic is astroturfing—orchestrating campaigns that appear grassroots-driven but are actually initiated by lobbyists. While potentially effective, astroturfing distorts perceptions of public opinion and undermines transparency and trust in advocacy (Walker and Le 2023).

Social proof operates through both fast and slow cognitive routes. Policymakers may deliberately assess public sentiment—whether factual or manufactured—through careful reasoning and evidence. In politics, unlike in consumer behavior where social proof is often seen as an irrational shortcut, following cues from public opinion may in fact be rational, as representation, legitimacy, and re-election depend on aligning with majority or constituency preferences. Yet complex reasoning is not required for social proof to be persuasive. Policymakers often respond to surface-level signals—media buzz, coalition names, or public-interest frames—that intuitively convey broad support but may prove misleading under closer scrutiny. As Walgrave and colleagues (2023) demonstrate, politicians are hardly better at estimating public preferences than ordinary citizens and often rely on superficial cues—media coverage, social media, or interest group claims—rather than data-driven opinion polls (Walgrave et al. 2023; Walgrave and Soontjens 2023). This suggests that heuristic, System 1 processing plays a substantial role in how politicians interpret social proof.

In practice, fast and slow processing of social cues are intertwined. Surface-level signals such as astroturf campaigns may capture attention and trigger immediate reactions while also prompting later scrutiny. Moreover, once such cues gain broad acceptance, they become political realities that policymakers must rationally account for. The key distinction is not whether a social cue is genuine or whether a reaction appears rational, but how the cue is processed in the moment. Fast, System 1 uptake occurs when signals prompt behavior without deliberate reflection. When this heuristic route dominates over deliberative reasoning, policymakers become particularly vulnerable to manufactured forms of social proof that exploit intuitive responses before conscious reflection can occur. Future research should therefore systematically investigate how lobbyists design and deploy social cues, how policymakers process them under varying conditions (e.g. uncertainty and similarity), and how these interactions ultimately shape policy outcomes and democratic legitimacy.

## Principle 6: consistency

Cialdini’s sixth principle of persuasion—consistency—is rooted in the psychological tendency of individuals to align their current behavior with past actions and commitments. Once a position is adopted, people experience both internal and social pressure to maintain it in order to appear reliable and coherent (Guadagno and Cialdini 2010). This principle is particularly relevant in lobbying, where policymakers operate under public scrutiny and are expected to act in accordance with their prior statements. As Brussels-based lobbyist Aaron McLoughlin puts it, consistency is “the most valuable” of Cialdini’s principles for lobbying.^14^

Consistency shapes lobbying strategies, particularly in target selection. Policymakers who have made strong public commitments opposing a lobbyist’s goals are less likely to shift positions, while those with aligned past positions are more receptive. While ideological alignment is often cited as a reason for targeting specific policymakers (Allern 2024; Bunea et al. 2025; Otjes and Rasmussen 2017), the consistency principle adds that such targets are also more predictable due to their motivation to remain consistent.

Lobbyists can also actively leverage consistency. Reminding individuals of prior commitments increases alignment (Cialdini and Trost 1998). In practice, lobbyists analyze past votes, speeches, and statements (De Bruycker and McLoughlin 2021) not only to identify which legislators or EU member states to target, but also to frame their requests as logical continuations of previously expressed positions.

Cialdini highlights that commitments made in public are particularly important in reinforcing consistency. Consequently the consistency principle also influences the choice between inside and outside lobbying. Inside lobbying—private engagement—allows lobbyists to influence policymakers before public commitments are made. Behind closed doors, there is less pressure for policymakers to remain consistent with past statements, and they are less likely to make binding commitments in public that contradict the organization’s goals. Additionally, inside lobbying gives lobbyists more control over how commitments are made or activated, as there are fewer external factors that might restrain their ability to sway decision-making. Outside lobbying, by contrast, can publicly remind policymakers of past commitments, reinforcing pressure to remain consistent. This dynamic helps explain why most lobby organizations prefer inside over outside lobbying (Binderkrantz 2005; Hanegraaff et al. 2016; Junk 2016).

Lobbyists may also encourage public appearances that subtly align policymakers with their interests. At a public seminar, a public affairs professional described an example involving the tobacco industry: a government official was invited to visit a site where counterfeit cigarettes had been seized.^15^ The event was presented as part of a broader campaign against illicit trade—a narrative often used to reframe regulatory debates in terms of enforcement rather than public health. Although the visit appeared neutral, it subtly positioned the minister in alignment with the industry’s framing. Such symbolic acts can create implicit commitments that shape future decisions, even in the absence of explicit policy endorsements.

Cialdini emphasizes that even small commitments can have lasting effects. He cites the example of American prisoners of war in the Korean War, who gradually adopted more sympathetic views toward communism after making minor concessions. Similarly, a policymaker who acknowledges a lobbyist’s perspective in a minor setting may become more aligned over time. Once a commitment is made, individuals tend to rationalize and reinforce it—what Cialdini calls “commitments growing their own legs.” This makes securing small initial commitments a cost-effective lobbying strategy.

Consistency also shapes long-term relationships. Once a policymaker has engaged with a lobbyist, continuing that relationship aligns with prior behavior. Sustained engagement fosters loyalty and collaboration. However, a rigid adherence to past strategies—engaging the same policymakers, supporting the same issues, and using the same lobbying tactics—can lead to strategic inertia. To remain effective, lobbyists must balance the power of consistency with adaptability, ensuring they do not fall into repetitive patterns that hinder innovation and responsiveness.

Consistency operates at the intersection of fast and slow lobbying. Slow lobbying leverages it through deliberate strategy: policymakers are reminded of past commitments to maintain credibility, while lobbyists frame new requests as logical extensions of prior positions. Fast lobbying, by contrast, taps into consistency as a cognitive shortcut. Under time pressure or cognitive overload, policymakers may default to earlier decisions without reassessment. The two modes can reinforce one another. Small symbolic acts—such as accepting a meeting or attending an event—can trigger fast, intuitive alignment that initiates incremental commitment. Over time, this may develop into deeper engagement with the lobbyist’s substantive ideas and policy evidence. Once a policymaker accepts a lobbyist’s position on one issue, they may become less scrutinizing on related files, aligning out of an impulse to remain consistent. Consistency thus enables influence without overt persuasion, gradually shifting preferences through a self-reinforcing feedback loop.

Despite its importance, consistency is underexplored—likely due to methodological challenges. Future studies could examine whether referencing past commitments enhances persuasion, how small commitments evolve into broader alignment, and how consistency influences target and strategy selection and ultimately lobbying access and influence.

## Principle 7: unity

Cialdini’s final principle—unity—posits that individuals are more likely to comply with requests from those they perceive as part of their in-group. Unlike liking, which is based primarily on interpersonal affection and positive feelings, unity centers on a shared collective identity—a sense of ‘we-ness’ rooted in common group membership, such as family, nationality, ethnicity, or political affiliation. This collective identity shapes behavior through tribal instincts and affective loyalty and is deeply embedded in self-conception. As Cialdini puts it, “the ‘we’ is the shared ‘me’.” He argues that when people perceive others as part of their group, they are more inclined to trust them, prioritize their welfare, and adopt their behaviors. This tribal instinct, according to Cialdini, forms part of the “click, run” response—an automatic, intuitive reaction that bypasses rational reflection.

Unity is particularly relevant in public affairs and lobbying, where shared identity can be a powerful lever of influence. Lobbyists often approach policymakers who share their national background or language—a common practice in Brussels (Bunea et al. 2025; De Bruycker 2024; Hollman and Murdoch 2018). Similarly, ideological alignment may foster more intensive exchanges, not necessarily due to shared policy goals, but because of a sense of belonging to the same political “tribe.” Even demonstrably inferior advice is trusted more when it comes from political allies (Marks et al. 2019; as cited in Cialdini 2021).

Beyond national and ideological alignment, friendship also reflects shared belonging. Unity helps explain why repeated cooperation between lobbyists and policymakers can lead to informal alliances or friendships. While friendship has been discussed under liking, reciprocity, and consistency, unity adds another layer: it is not just about affection, but about shared identity. When lobbyists and policymakers work together repeatedly—on committees, in forums, or at conferences—they may begin to see each other as part of the same team. This sense of “we-ness” can make policymakers more inclined to support a lobbyist’s position—not because it is objectively superior, but because it comes from someone perceived as one of their own.

In rare cases, family ties may also matter. For example, Politico^16^ reported that Roberta Metsola’s husband, a senior lobbyist for Royal Caribbean Group, received unusually prompt replies from EU officials while she was Parliament president. While such cases are exceptional, they illustrate how personal relationships can intersect with lobbying strategies, raising broader questions about transparency and democratic accountability.

When shared identities are absent, lobbyists may seek to create unity through shared activities—such as social events at international summits, thematic conferences, or informal clubs. These gatherings are often organized to cultivate a sense of belonging and collective identity among participants. or example, a recent *Politico* feature, *“How the Gym Became Brussels’ Elite New Lobbying Spot*,*”* 17 vividly illustrates this phenomenon: high-end gyms in Brussels now serve as informal networking arenas where lobbyists, EU officials, and journalists build professional ties through shared fitness routines. These spaces foster bonds grounded in perceived similarity and shared lifestyle rather than formal institutional connection. As one consultant noted, “sooner or later, the people you meet doing sports might become a professional contact—whether you want it or not.” Such environments blend the personal and professional: engaging in the same activities, at the same place and time, reinforces a sense of “we-ness,” which may later facilitate a lobbyist’s influence.

Another example is the European Parliament Beer Club^18^ (recently rebranded as the European Beer Group), a group of Members of the European Parliament sympathetic to the brewing industry who offers their members a *“unique opportunity to unite and stay informed on beer-related issues through various activities”*. Shared group activities—such as informal clubs or gyms—whether social, professional, or symbolic—can foster solidarity and mutual identification. Neuroscientific research cited by Cialdini shows that people engaged in joint tasks exhibit synchronized brainwave patterns, and behavioral studies find that synchronized movement (e.g., marching, smiling) increases cooperation (e.g., Cappella 1997). Likewise, in lobbying such shared experiences may subtly shift a policymaker’s perception of a lobbyist from outsider to insider.

Some interest groups strategically leverage unity to shape policy debates. For example, Sanchez Salgado (2021) shows how, during EU asylum reform debates, actors in the European Parliament used emotional frames centered on solidarity and compassion for refugees to counter fear-based narratives. Similarly, Baumgartner et al. (2008) describe how the morality frame in the U.S. death penalty debate was gradually replaced by an innocence frame—highlighting wrongful convictions and fostering identification with those on death row.

From a theoretical perspective, unity aligns closely with fast lobbying. It operates through intuitive, emotional, and automatic processes rather than deliberative reasoning. While unity can enhance information exchange by fostering trust, its primary power lies in triggering affective responses rooted in tribalism. When lobbyists organize shared activities or build reciprocal relationships, they are not merely networking—they are cultivating a shared identity. This sense of “we-ness” can make policymakers more inclined to say yes—not because the request is rationally optimal, but because it comes from someone perceived as one of us.

However, unity often develops through the longer-term relationship-building characteristic of slow lobbying. Sustained interactions, collaborative efforts, and trust built over time can foster group identification, prompting policymakers to see lobbyists as insiders. Once established, this shared identity not only triggers intuitive acceptance but also makes policymakers more receptive to the lobbyist’s arguments and evidence. Unity thus exemplifies how fast and slow lobbying are deeply intertwined and mutually reinforcing: affective responses may emerge from slow relational work, while fast, intuitive trust can lay the groundwork for more reflective engagement.

## Conclusion

This research agenda article explored an underexamined dimension of lobbying: its affective, relational, and psychological underpinnings. While interest group literature has largely portrayed lobbying as a rational, transactional exchange between organizations and policymakers (Bunea and Baumgartner 2014, p. 10), this article argues for a broader conceptualization—one that accounts for emotionally driven and intuitive forms of influence. It responds to two key gaps: the dominance of rationalist models in interest group research and the limited integration of behavioral and psychological insights into lobbying theory.

To address these gaps, the article introduced the distinction between “fast” and “slow” lobbying, modeled on dual-process theories of decision-making. Drawing on Daniel Kahneman’s work on cognitive processing and Robert Cialdini’s principles of persuasion, it proposed a conceptual framework to theorize how influence operates through both deliberative and intuitive routes. While these insights have been widely used in marketing and psychology, this article repurposed them to theorize lobbying as a dual-process phenomenon. This conceptual reframing bridges micro-level behavioral mechanisms with meso- and macro-level patterns of political influence. By doing so, it brings Kahneman’s and Cialdini’s work into dialogue with institutional and normative questions about representation, access, and democratic legitimacy—questions that are typically absent from the behavioral sciences but central to interest group research.

This agenda-setting piece did not aim to test specific hypotheses, but to spark future research into the role of bounded rationality, psychological biases, and informal influence in public affairs. It proposes that lobbying strategies aligned with fast thinking—such as appeals to familiarity, likability, and in-group unity—may have underappreciated effects on elite decision-making. The article calls for more attention to how lobbying shapes policymakers’ intuitive responses. It invites scholars to expand the lobbying research toolkit and explore the ethics of subconscious persuasion in democratic settings.

**Table 1:** Fast and slow lobbying across cialdini’s seven principles

Principle	Fast Lobbying (System 1)	Slow Lobbying (System 2)
Reciprocity	Leveraging symbolic gifts and favors to trigger reciprocal obligation.	Exchanging expertise to meet policymakers’ informational needs.
Scarcity	Rhetorically creating urgency or fear of loss to prompt quick decisions.	Supporting urgency claims with evidence and analytical justification.
Liking	Building rapport through friendship, similarity, charm and personal praise.	Cultivating long-term relationships to support expertise-driven exchanges.
Social Proof	Framing majority opinion to trigger heuristic action and conformity.	Informing policymakers about public sentiment to support reasoned judgment.
Authority	Invoking titles, endorsements, or perceived expertise to gain compliance.	Providing substantive expertise and building credibility over time.
Consistency	Securing small symbolic commitments that prompt future compliance and gradually deepen alignment.	Analyzing and communicating past votes, behavior and statements to help policymakers act consistently.
Unity	Cultivating a shared identity through social activities and emotional framing.	Cultivating shared identity and trust through sustained collaboration.

Table 1 outlines how Cialdini’s principles operate across fast and slow lobbying. Each row points to an observable mechanism that can be operationalized and analyzed. In particular, the left column—focused on fast lobbying—offers a fertile ground for further empirical inquiry. Researchers might, for instance, examine whether symbolic gestures such as small gifts (reciprocity) influence access outcomes, or how emotional rapport and perceived similarity (liking and unity) affect the credibility or uptake of policy proposals. Future research could test which principles dominate at different policymaking stages or across formal vs. informal channels.

This perspective shifts the analytical focus from organizational-level transactions to the interpersonal dynamics between individual lobbyists and policymakers. While democratic institutions are designed to filter out the psychological biases discussed in this paper, the individuals within these institutions remain susceptible to cognitive limitations and emotional influences. Scholars are therefore encouraged to examine how micro-level encounters—such as similarity or friendship—shape access and receptiveness. Although policymaking often involves multiple actors and systems of checks and balances, this does not preclude psychological biases from playing a role. For instance, research shows that European Commissioners often recruit cabinet staff who share their national background (Egeberg 2010), which may in turn result in increased interactions with lobbyists of similar backgrounds.

The presentation of these two modes of lobbying highlights promising directions for further exploration in interest group research. First, a key blind spot identified in this article is the portrayal of lobbying as purely technocratic or as a transmission belt between citizens and policymakers (Albareda and Braun 2019; Berkhout 2013). While this approach may enhance the perceived legitimacy of lobbying—by framing it as a contribution to informed and responsible decision-making—it simultaneously obscures practices that fall outside this lens. When research focuses narrowly on exchanges of information, it risks overlooking influential dynamics such as friendship, deceptive framing, and in-group favoritism. These informal, relational, and often opaque practices may not be absent from lobbying—they may simply remain unobserved because they do not fit into the dominant transactional framework. As such, scholars may need to reevaluate the legitimacy and democratic implications of lobbying in light of more fluid and less accountable techniques. This also offers opportunities for those studying lobbying ethics and regulation (Chari et al. 2019; Năstase 2020) to conceptualize new codes of conduct and oversight regimes that account for fast lobbying dynamics more explicitly.

Second, the article calls for a deeper integration of psychological and communication theories and methods into lobbying research. Disciplines such as psychology have long acknowledged the cognitive biases that shape human behavior and the persuasive opportunities they create. Theories such as prospect theory, similarity attraction, and reactance theory offer valuable frameworks for predicting how policymakers may respond to influence attempts. This shift calls for a reorientation of what and how we study influence, rather than a full methodological overhaul. Methodologically, a shift toward more experimental research to tease out psychosocial mechanisms and effects—alongside the use of longitudinal research designs to observe relational patterns over time, and biographical data to account for individual characteristics—would be a logical next step.

Finally, the article draws attention to a new form of bias that centers on the human resources available to interest groups. Lobbying success may be shaped not only by formal expertise or organizational capacity, but also by the social capital, charisma, and communicative skills of individual lobbyists. Biases may favor lobbyists who share identities or networks with policymakers. These dynamics echo the concerns raised by Mills (1959) in his theory of the “power elite,” where political and economic elites are bound together by shared backgrounds and informal social ties. Future research should explore whether similar elite formations exist in lobbying and public affairs, and how these shared traits and informal ties shape access and influence.

While Cialdini’s principles are useful and encompassing, they do not capture all relevant factors. Other psychological mechanisms —such as present-oriented biases (e.g., hyperbolic discounting), memory-based distortions like the peak-end rule, anchoring effects, status quo bias, and availability heuristics—may also shape lobbying outcomes (e.g., Benhabib et al. 2010; Kahneman 2011; Thaler and Sunstein 2021). While Cialdini’s seven principles offer a solid foundation, future research should remain open to identifying additional pathways of influence to develop a more comprehensive and nuanced understanding of fast lobbying and its interplay with slow, evidence-based approaches.

In sum, while this article offers a comprehensive overview, it only begins to uncover how cognitive biases among policymakers may be leveraged by lobbyists. Still, it makes a strong case for recognizing the importance of intuitive influence techniques in shaping policymaker behavior. These techniques may carry far-reaching implications for how interest groups shape public policy—and for how we understand political influence in democratic systems.

## Data Availability

No datasets were generated or analysed during the current study.

## Footnotes

1
https://www.knack.be/nieuws/uberfiles-achter-de-coulissen-van-het-brusselse-taxiplan/.

2
https://www.politico.eu/article/roberta-metsola-wine-eu-parliament-president-lavish-dinner-free-hotel-le-cep-stay-revealed-confrerie-des-chevaliers-du-tastevin.

3
https://www.politico.eu/article/eropean-union-parliament-president-roberta-metsola-champagne-earphones-gold-gift-declare-qatargate-corruption-lobbying-antonio-panzeri-eva-kaili/.

4
https://www.transportenvironment.org/about-us.

5
https://www.businesseurope.eu/publications/businesseurope-welcomes-draghi-report-and-calls-urgent-action-restore-eus-competitive; https://www.europen-packaging.eu/news/the-draghi-report-unpacked/.

6
https://www.ft.com/content/8be1a975-e5e0-417d-af51-78af17ef4b79.

7 e.g. https://www.politico.eu/article/michel-escapes-disciplinary-action-over-amendments-to-data-rules/.

8
https://www.businesseurope.eu/wp-content/uploads/2025/02/2022-10-14_businesseuropes_message_to_the_european_council_on_20-21_october-621-1.pdf.

9
https://www.businesseurope.eu/publications/new-study-shows-that-europes-energy-price-gap-will-worsen-without-urgent-action/.

10 Author’s notes, informal conversation with EU official, 2022.

11
https://time.com/archive/6596688/jack-abramoff-the-man-who-bought-washington/.

12
https://www.greenpeace.org/usa/jane-fonda-jason-momoa-joaquin-phoenix-and-other-celebrities-call-on-biden-administration-to-support-a-strong-plastics-treaty-ahead-of-negotiations-in-paris/.

13
https://time.com/5197255/facebook-cambridge-analytica-donald-trump-ads-data/.

14
https://www.aaronmcloughlin.com/5704-2/.

15 Author’s notes from public seminar, 2016.

16
https://www.politico.eu/article/roberta-metsola-lobbyist-husband-ukko-european-parliament-president-transparency/.

17
https://www.politico.eu/article/gym-brussels-new-networking-power-lobbying-spot-aspria-basic-fit-animo/.

18 https://beergroup.eu.
